# SARS-CoV-2 epitope-specific CD4^+^ memory T cell responses across COVID-19 disease severity and antibody durability

**DOI:** 10.1126/sciimmunol.abl9464

**Published:** 2022-04-21

**Authors:** Ryan W. Nelson, Yuezhou Chen, Olivia L. Venezia, Richard M Majerus, Daniel S. Shin, MGH COVID-19 Collection & Processing Team, Mary N. Carrington, Xu G. Yu, Duane R. Wesemann, James J. Moon, Andrew D. Luster

**Affiliations:** ^1^ Division of Immunology, Boston Children’s Hospital, Harvard Medical School; Boston, MA, USA.; ^2^ Center for Immunology and Inflammatory Diseases, Division of Rheumatology, Allergy and Immunology, Massachusetts General Hospital, Harvard Medical School; Boston, MA, USA.; ^3^ Department of Medicine, Division of Allergy and Clinical Immunology, Division of Genetics, Brigham and Women’s Hospital, Harvard Medical School; Boston, MA, USA.; ^4^ Queens University of Charlotte, Charlotte, NC, USA.; ^5^ Ragon Institute of MGH, MIT and Harvard; Cambridge, MA, USA.; ^6^ Basic Science Program, Frederick National Laboratory for Cancer Research, National Cancer Institute, Frederick, MD and Laboratory of Integrative Cancer Immunology, Center for Cancer Research, National Cancer Institute; Bethesda, MD, USA.; ^7^ Infectious Disease Division, Brigham and Women’s Hospital, Harvard Medical School; Boston, MA, USA.; ^8^ Division of Pulmonary and Critical Care Medicine, Massachusetts General Hospital, Harvard Medical School; Boston, MA, USA.

## Abstract

CD4^+^ T cells are central to long-term immunity against viruses through the functions of T helper-1 (Th1) and T follicular helper (Tfh) cell subsets. To better understand the role of these subsets in COVID-19 immunity, we conducted a longitudinal study of SARS-CoV-2-specific CD4^+^ T cell and antibody responses in convalescent subjects who seroconverted during the first wave of the pandemic in Boston, Massachusetts, United States, across a range of COVID-19 disease severities. Analyses of spike (S) and nucleocapsid (N) epitope-specific CD4^+^ T cells using peptide and major histocompatibility complex class II (peptide:MHCII) tetramers demonstrated expanded populations of T cells recognizing the different SARS-CoV-2 epitopes in most subjects compared to pre-pandemic controls. Individuals who experienced a milder disease course not requiring hospitalization had a greater percentage of circulating Tfh (cTfh) and Th1 cells among SARS-CoV-2-specific cells. Analysis of SARS-CoV-2-specific CD4^+^ T cells responses in a subset of individuals with sustained anti-S antibody responses following viral clearance also revealed an increased proportion of memory cTfh cells. Our findings indicate efficient early disease control also predicts favorable long-term adaptive immunity.

## Teaser

The proportion of cTfh within SARS-CoV-2-specific cells was decreased in severe disease and increased in sustained antibody producers.

## INTRODUCTION

The adaptive immune system can provide strong and durable cellular and humoral immunity to viral infections, such as SARS-CoV-2, through coordinated T and B cell responses. CD4^+^ “helper” T cells play a central role as they differentiate into T helper type 1 (Th1) cells, to stimulate phagocytes and cytotoxic CD8^+^ T cells, and T follicular helper (Tfh) cells to promote high affinity and long-lived antibody responses by B cells in germinal center (GC) reactions. This normally occurs in a highly coordinated fashion beginning with early innate immune sensing of viral infection and propagating signals that lead to T cell activation, differentiation, and protective memory cell formation. However, disruptions in innate immune functions are well documented through the course of the COVID-19 pandemic and at least partially explain the broad outcomes of SARS-CoV-2 infection in humans. The clearest example is immune deficiencies of type I interferon (IFN-I) responses through inborn errors (*1*) or autoantibodies (*2*), which are causally linked to at least 13.7% of life-threatening acute SARS-CoV-2 infections.

Factors associated with acute disease severity are associated with variability in early T and B cell responses across numerous patient cohorts. It is less clear how variability in early T and B cell responses impacts long-term immunity. Studies early in the pandemic showed high peak SARS-CoV-2-specific antibody titers particularly in severe cases of COVID-19. However, these appeared to quickly decay, suggesting humoral protection may be short-lived in some cases (*3*, *4*). Subsequent longitudinal analyses show relatively stable memory T and B cell responses over time following mild (*5*–*7*) and even asymptomatic (*8*) infections. Among individuals with mild symptoms that did not require hospitalization, those with the shortest symptom duration had more sustained antibody levels and increased somatic hypermutation (*9*). This antibody ‘sustainer’ phenotype observed during mild COVID-19 suggests a greater contribution of GC-dependent B cell responses and more efficient generation of long-lived plasma cells compared to individuals whose SARS-CoV-2-specific antibodies decayed. Furthermore, post-mortem lymph node and spleen analysis of fatal acute COVID-19 revealed near complete absence of germinal centers (*10*). Thus, the quality and durability of B cell responses to SARS-CoV-2 appear related to early disease control.

While there are fewer studies focused on the clinical correlates of T cell responses to SARS-CoV-2, some reports during acute infections found evidence for greater T cell response magnitude in severe COVID-19 (*11*, *12*), whereas others found IFN-γ-producing SARS-CoV-2-specific Th1 and CD8^+^ T cell responses (*13*, *14*), as well as cTfh cells (*13*), were inversely correlated with disease severity during acute COVID-19. Discrepancies in measured T cell responses could be related to differences in the methods used for detecting SARS-CoV-2-specific T cell populations. This has been approached largely by indirect assessment of markers associated with recent T cell activation or by measuring cytokine production capacity or activation induced marker (AIM) expression upon restimulation of T cells with SARS-CoV-2-expressed peptide pools ex vivo.

To better understand immune memory to COVID-19, we generated SARS-CoV-2-derived peptide bound major histocompatibility complex II (pMHCII) tetramers to directly quantify and track CD4^+^ T cell responses at the level of individual spike (S)- and nucleocapsid (N)-epitope-specific cells in their native state without reactivation. We performed this analysis in convalescent subjects with a range of COVID-19 disease severities during the first year following infection. Circulating N- and S-epitope-specific CD4^+^ memory T cells were detected in most individuals for the duration of the study. Following mild COVID-19, these were largely characterized by phenotypically stable Th1 or cTfh cells. In a portion of these subjects, increased memory cTfh responses also correlated with sustained antibody responses over time. This contrasted with subjects previously hospitalized with moderate to severe symptoms who had decreased percentages of Th1 and Tfh phenotype circulating memory cells, which correlated poorly with antibody responses. These findings demonstrate the utility of directly assessing SARS-CoV-2-specific CD4^+^ T cells with pMHCII tetramers and suggest immune dysregulation associated with severe COVID-19 impacts the quality of T and B cell memory.

## RESULTS

### SARS-CoV-2 epitope identification and patient cohorts

We predicted CD4^+^ T cell epitopes for multiple HLA-DR alleles using publicly available peptide-MHC binding algorithms (*15*, *16*), and cross-referenced them with peptide sequences reported to be immunodominant in earlier studies (*17*–*21*) (Table S1). We generated a panel of 37 tetramers across 7 common HLA-DR alleles and screened peripheral blood mononuclear cells (PBMC) from at least 2 HLA-matched COVID-19 convalescent subjects per tetramer. PBMC were stained with fluorochrome-labeled tetramers and magnetically enriched as previously described (*22*). The general gating strategy and results of epitope identification using pMHCII tetramers are presented in Supplemental Fig. 1. We identified two non-overlapping spike (S166-177 and S310-320) and two non-overlapping nucleocapsid (N305-316 and N329-340) peptides that each bound the prevalent HLA-DRB1*07:01 (DR7) allele and detected responses in subjects with known SARS-CoV-2 infection history. We focused our study on DR7^+^ subjects given the increased number of individual epitopes identified with our tetramer-based approach, and increased availability of samples due the high prevalence of this allele (25%) in our study population.

PBMC and plasma samples from 40 DR7^+^ convalescent subjects enrolled by the Massachusetts Consortium on Pathogen Readiness longitudinal cohort were analyzed (MassCPR cohort, Table S2). All convalescent subjects had confirmed infection by SARS-CoV-2 PCR or antibody detection, and convalescent samples were obtained beginning after symptom resolution and/or following negative repeat PCR. One to three blood samples per subject were obtained between April 2020 and January 2021 (ranging from 13 to 333 days after symptom onset). Nine of the 40 subjects were previously hospitalized for moderate to severe acute COVID, and 6 of the 9 subjects also required admission to the intensive care unit (ICU). Three hospitalized subjects received immunomodulatory treatments – 2 received methylprednisolone and 1 received tocilizumab. No subjects received anti-SARS-CoV-2 monoclonal antibodies. The remaining non-hospitalized subjects were managed at home with milder symptoms. Additional demographic information is found in Table S2.

We also analyzed PBMC and plasma from 21 additional convalescent subjects from a Brigham and Women’s Hospital (BWH) cohort, obtained between August and November 2020 (ranging from 150 to 242 days after symptom onset) for SARS-CoV-2 epitope-specific CD4^+^ T cells (Table S2). These subjects were identified as DR7^+^ from this previously described cohort (*9*), and all had mild symptoms not requiring hospitalization. Our measured T cell and antibody responses at these time points were paired with the previously reported antibody measurements from each study subject to determine long term durability.

### Detection of SARS-CoV-2-specific CD4^+^ T cell responses using combinatorial tetramer staining

**Fig. 1.**
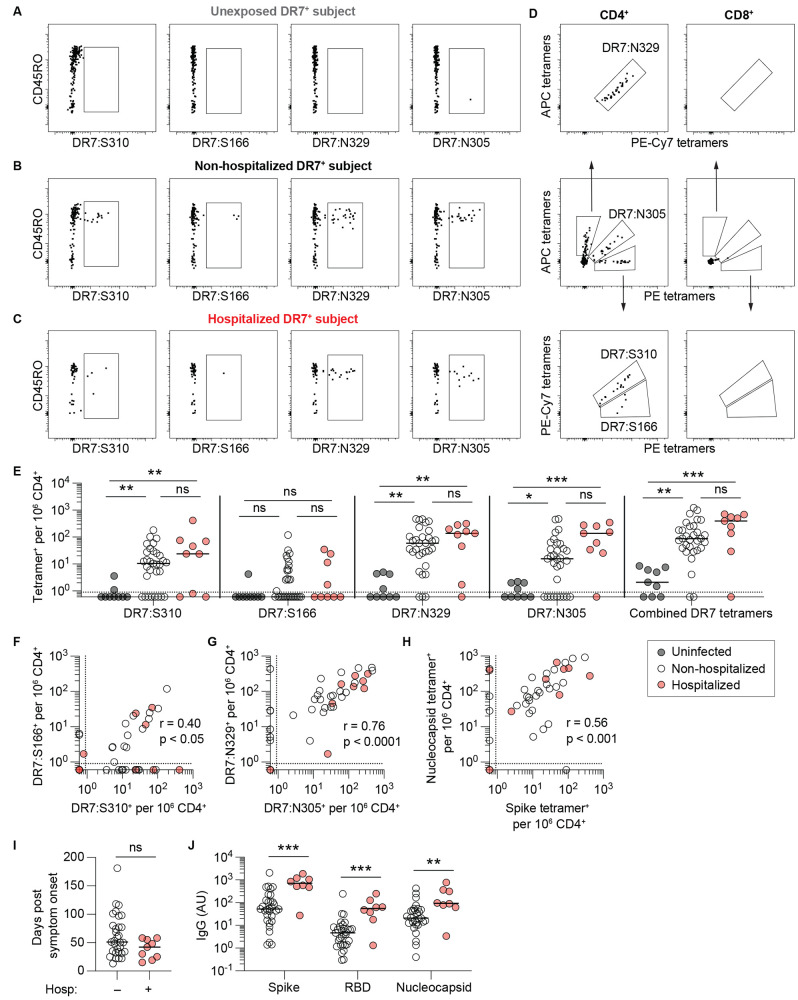
Detection of SARS-CoV-2-specific CD4^+^ T cell responses using combinatorial tetramer staining. PBMC from DRB1*07:01 (DR7)^+^ subjects were stained with DR7-matched spike and nucleocapsid tetramers, magnetically enriched, and analyzed by flow cytometry. (**A-C**) Representative flow cytometry plots of live CD14^–^CD20^–^CD3^+^CD8^–^CD4^+^ gated events with indicated tetramer^+^ gates for the 4 epitope-specific populations from a pre-pandemic negative control (**A**), a non-hospitalized subject (**B**), and a previously hospitalized subject (**C**). (**D**) Representative flow cytometry plots of combinatorial tetramer staining of live CD14^–^CD20^–^CD3^+^CD8^–^CD4^+^ gated events (left column) and live CD14^–^CD20^–^CD3^+^CD8^+^CD4^–^ gated events (right column). (**E**) Summary of individual epitope-specific CD4^+^ T cell frequencies and combined tetramer^+^ cells per million CD4^+^ T cells. Uninfected n = 9, non-hospitalized n = 31, previously hospitalized n = 9. Statistics by Kruskal-Wallis and Dunn’s multiple comparisons tests. (**F-H**) Correlations between DR7:S310^+^ and DR7:S166^+^ (spike-specific) cell frequencies (**F**), between DR7:N305^+^ and DR7:N329^+^ (nucleocapsid-specific) cell frequencies (**G**), and between nucleocapsid-specific (combined DR7:N305^+^ and DR7:N329^+^) and spike-specific (combined DR7:S310^+^ and DR7:S166^+^) cell frequencies (**H**), with r and significance from Spearman correlation. (**I**) Comparison of timing of first time point sample collection of all non-hospitalized and previously hospitalized subjects in the MassCPR cohort. (**J**) Summary of circulating anti-S, anti-RBD, and anti-N IgG antibody levels from paired plasma samples. AU denotes arbitrary units. Statistics by Mann-Whitney tests. Solid horizontal lines indicate median values. Dotted horizontal line indicates limit of detection. *p < 0.05, **p < 0.01, ***p < 0.001. ns = not statistically significant.

To determine the magnitude of early CD4^+^ memory T cell responses to SARS-CoV-2 infection, we initially assessed SARS-CoV-2-specific CD4^+^ T cell responses in convalescent subjects at the first time point of sample collection of all 40 DR7^+^ convalescent subjects. We also tested cryopreserved PBMC from 9 DR7^+^ individuals drawn before December 2019 as SARS-CoV-2-unexposed negative controls. Representative flow cytometry plots of uninfected, non-hospitalized, and previously hospitalized subjects are shown in Figs. 1A-C. We detected expanded populations of spike (DR7:S310 and DR7:S166)- and nucleocapsid (DR7:N329 and DR7:N305)-specific cells within the CD4^+^ T cell compartment of most non-hospitalized and previously hospitalized subjects. CD4^+^ T cells from COVID-19 subjects that recognized these epitopes expressed CD45RO, a marker of antigen experience (Fig. 1A-C).

A combinatorial tetramer staining strategy was employed, which enabled magnetic enrichment for all four cell populations within a single sample using a combination of PE, APC and PE-Cy7 fluorophores (Fig. 1D). Tetramer staining was TCR-specific and restricted to CD4^+^ T cells, with negligible background staining of CD8^+^ T cells. We set the limit of detection based upon the mean frequency of CD8^+^ T cells (0.9 per million CD8^+^ T cells (Fig. S2). The lowest detectable cell frequency for any tetramer was 0.6 per million CD4^+^ T cells. We analyzed subjects separated based upon whether they had mild disease not requiring hospitalization (WHO classification groups 1-2) or moderate to severe disease requiring hospitalization (WHO groups 3-7) to approximate disease severity. We found more CD4^+^ memory T cells recognizing DR7:S310, DR7:N329, and DR7:N305 per million circulating CD4^+^ T cells in both non-hospitalized and previously hospitalized subjects compared to uninfected controls (Fig. 1E). DR7:S166 was the weakest epitope in convalescent subjects. We did not find a statistical difference in cell frequencies between either convalescent subject group and the uninfected controls. There was a trend toward increased frequency of tetramer detected cells in previously hospitalized subjects compared to non-hospitalized subjects, with higher median cell frequency for DR7:S310, DR7:N329 and DR7:N305-specific cells, though these were not statistically significant differences. CD4^+^ T cells recognizing at least 1 of the 4 epitopes were also identified in 6 out of 9 uninfected controls at low frequencies. However, 90% (36 of 40) of DR7^+^ convalescent subjects had an increased frequency of SARS-CoV-2 tetramer bound cells as compared to the highest detectable frequency in uninfected controls. The magnitude of the response within each individual subject also correlated across the 4 tetramers in both non-hospitalized and previously hospitalized groups (Fig. 1F-H).

We simultaneously analyzed paired plasma samples for anti-S, anti-RBD, and anti-N IgG levels in DR7^+^ individuals in the MassCPR convalescent COVID-19 cohort at the first time point of collection, which ranged from 13 to 181 days after symptom onset (Fig. 1I). In contrast to SARS-CoV-2-specific CD4^+^ T cell responses (Fig. 1E), there were significant differences in early antibody responses based upon disease severity (Fig. 1J). Previously hospitalized patients had higher anti-S, anti-RBD, and anti-N IgG levels as compared to non-hospitalized patients at early convalescent time points, similar to previous reports (*4*, *23*–*25*).

### Kinetics of circulating SARS-CoV-2-specific CD4^+^ T cell responses in convalescent subjects

**Fig. 2.**
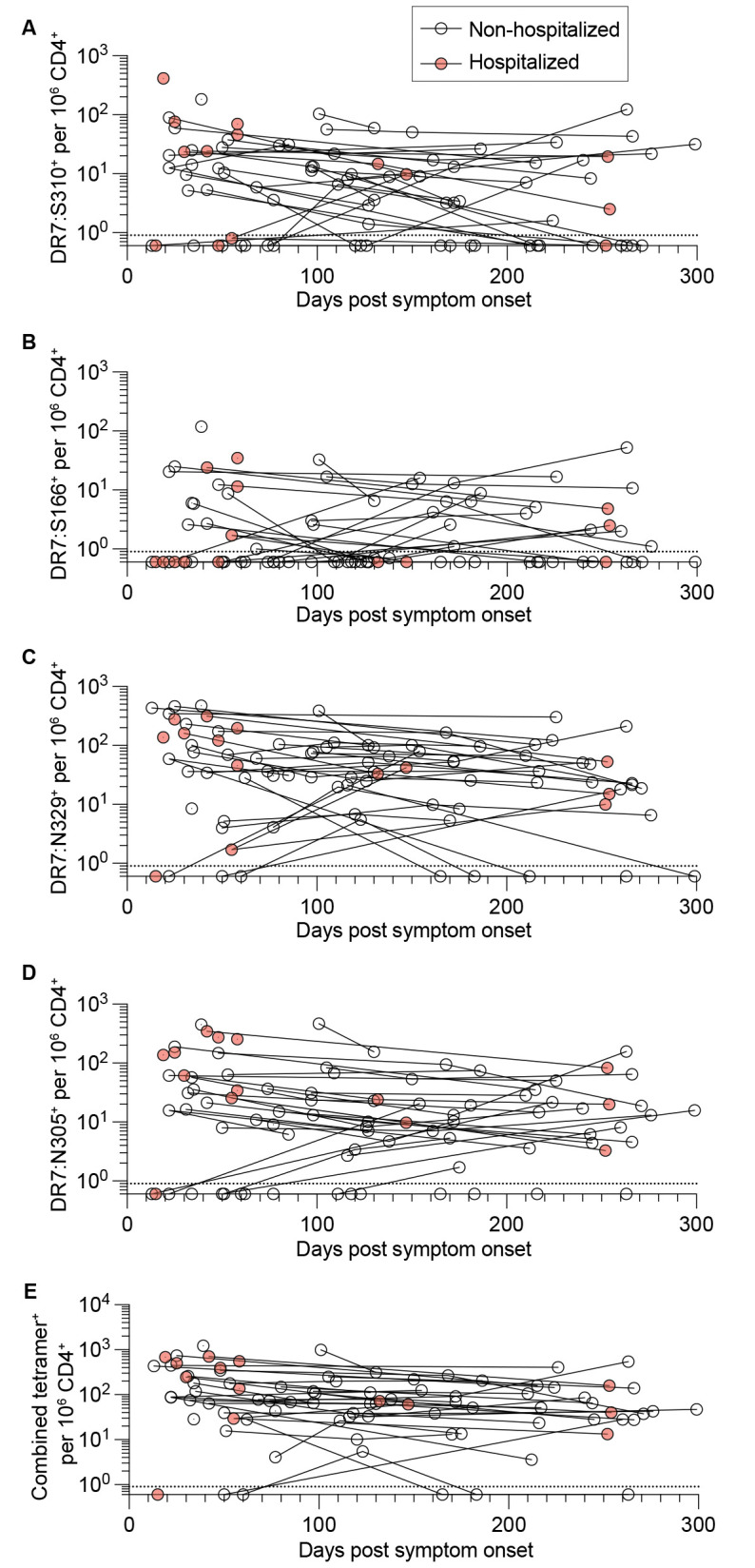
Kinetics of circulating SARS-CoV-2-specific CD4^+^ memory T cell responses in convalescent subjects. (**A-E**) Longitudinal analysis of tetramer^+^ CD4^+^ T cell frequencies per million CD4^+^ T cells over time for DR7:S310^+^ (**A**), DR7:S166^+^ (**B**), DR7:N329^+^ (**C**), DR7:N305^+^ (**D**), and combined tetramer^+^ (**E**) populations. Lines connect datapoints from the same subjects. Dotted horizontal line indicates limit of detection.

We analyzed longitudinal samples to determine the stability of CD4^+^ T cell memory to spike and nucleocapsid epitopes over a period of up to 10 months. This analysis was predominantly from non-hospitalized subjects due to fewer available samples from previously hospitalized subjects. The rates of linear decay of SARS-CoV-2-specific cells per million CD4^+^ T cells detected with each tetramer were not statistically different. We calculated the average half-life of linear decay from longitudinal subjects and observed similar kinetics across the 2 spike (DR7:S310^+^ *t*_1/2_ of 148 days and DR7:S166^+^ *t*_1/2_ of 135 days) and 2 nucleocapsid (DR7:N329^+^ *t*_1/2_ of 122 days and DR7:N305^+^ *t*_1/2_ of 165 days) epitopes (Fig. 2A-E). These half-life values were similar to previously reported SARS-CoV-2-specific CD4^+^ T cell memory stability using activation induced markers for detecting virus-specific cells (*5*).

### Kinetics of anti-SARS-CoV-2 spike (S), receptor-binding domain (RBD), and nucleocapsid (N) IgG antibody responses

**Fig. 3.**
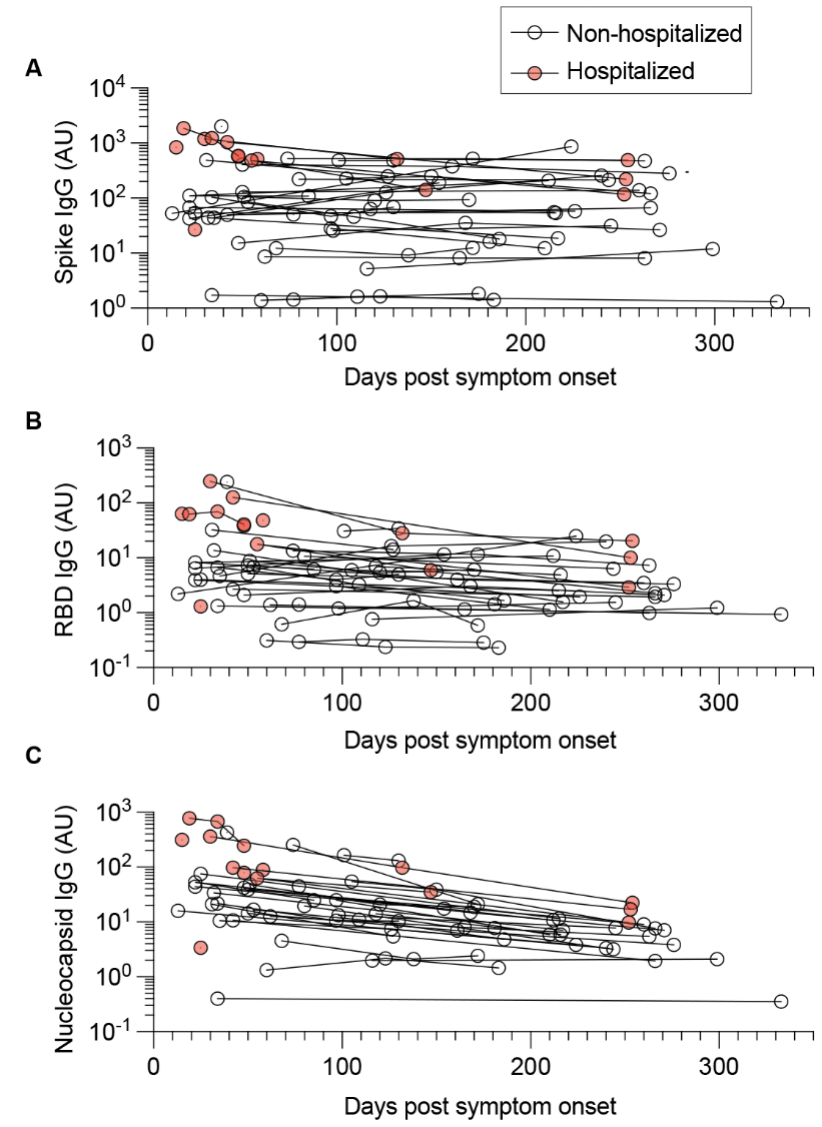
Kinetics of anti-SARS-CoV-2 spike (S), receptor-binding domain (RBD), and nucleocapsid (N) IgG antibody responses. Spike, RBD, and nucleocapsid IgG levels were measured by ELISA. (**A-C**) Longitudinal analysis of circulating antibodies over time, including anti-S IgG (**A**), anti-RBD IgG (**B**), and anti-N IgG (**C**). Lines connect datapoints from the same subjects. AU denotes arbitrary units.

We also observed differences in the durability of antibodies targeting the S, RBD, and N antigens. Anti-S responses were the most stable (Fig. 3A), followed by anti-RBD (Fig. 3B) and anti-N (Fig. 3C), as reported by others (*5*, *9*). Among individuals where two or more longitudinal plasma draws were obtained, ~ 44% (14 of 32) had sustained anti-S antibodies. Sustained antibody responses were defined by a final time point IgG level, drawn at least 4 weeks after the initial timepoint, at or above the initial level. The average duration of time between measurements was 163 days. Sustained anti-RBD IgG responses were also detected in some (8 of 32) individuals, whereas only 2 of 32 had sustained anti-N IgG responses in our study cohort.

### CD4^+^ T cell phenotypic differences between non-hospitalized and previously hospitalized patients

**Fig. 4.**
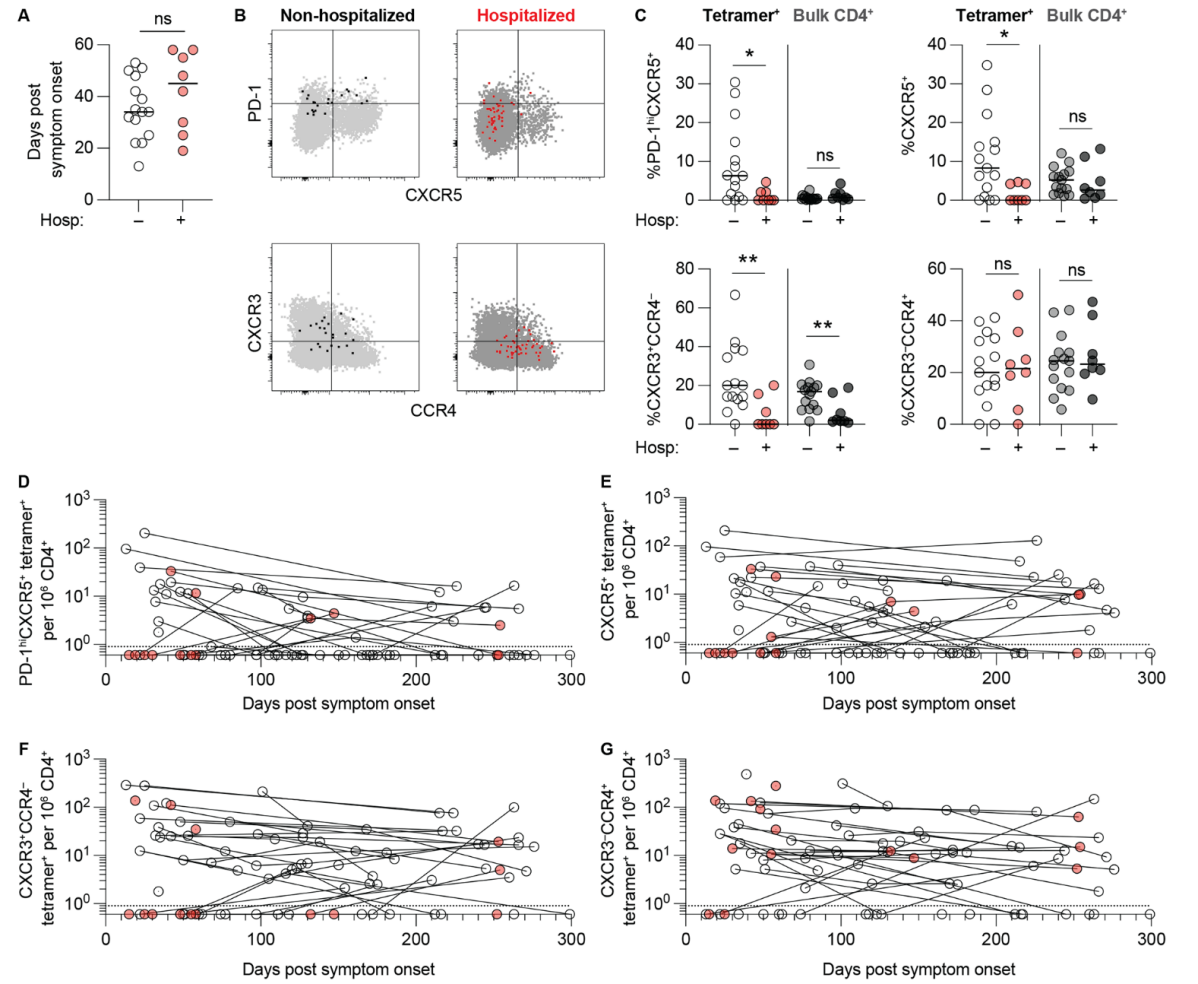
CD4^+^ T cell phenotypic differences between non-hospitalized and previously hospitalized patients. (**A**) Comparison of timing of sample collection of non-hospitalized (n = 15) and previously hospitalized (n = 8) subjects for phenotypic comparisons. (**B**) Representative PD-1 x CXCR5 staining (top row) and CXCR3 x CCR4 staining (bottom row) of live CD4^+^ T cell events. The left column represents an example of a non-hospitalized subject with combined tetramer^+^ (bound fraction, black) and tetramer^–^ bulk CD4^+^ (unbound fraction, light gray) gated cells. The right column represents a previously hospitalized subject with combined tetramer^+^ (bound fraction, red) and tetramer^–^ bulk CD4^+^ (unbound fraction, dark gray) gated cells. (**C)** Percentages of PD-1^hi^CXC5^+^, CXCR5^+^, CXCR3^+^CCR4^–^, and CXCR3^–^CCR4^+^ cells within combined tetramer^+^ and bulk (tetramer^–^) CD4^+^ T cells from non-hospitalized and previously hospitalized subjects. Horizontal lines indicate median values. Statistics by Mann-Whitney tests. *p < 0.05, **p < 0.01. ns = not statistically significant. (**D-G**) Scatter plots illustrating changes in combined tetramer^+^ PD-1^hi^CXC5^+^ (**D**), CXCR5^+^ (**E**), CXCR3^+^CCR4^–^ (**F**), and CXCR3^–^CCR4^+^ (**G**) cells per million CD4^+^ T cells over time. Lines connect datapoints from the same subjects.

To explore the possibility that acute disease severity affected the quality of SARS-CoV-2-specific CD4^+^ T cell responses, we characterized the phenotypes of circulating SARS-CoV-2-specific CD4^+^ memory T cells. Given strong correlations between the magnitude of individual DR7:S- and DR7:N-tetramer^+^ CD4^+^ T cell responses (Fig. 1F-H), we pooled responses to our 4 tetramers to increase overall cell number and statistical power for this analysis. To control for timing of infection, we analyzed samples within 60 days of symptom onset, which was the latest first blood draw for hospitalized subjects (Fig. 4A).

Cells were defined by chemokine receptor expression, with Th1 cells expressing CXCR3 but lacking CCR4 and Tfh cells expressing CXCR5 (Fig. 4B). PD-1 co-expression on some CXCR5^+^ tetramer^+^ cells identified a subset of activated cTfh that may be more abundant in acute compared to convalescent COVID-19 (*13*) and is also described early following live viral vaccination (*26*). Non-hospitalized subjects largely produced a mixed phenotype consisting of SARS-CoV-2-specific CXCR5^+^ memory cTfh cells, some PD-1^hi^CXCR5^+^ activated cTfh phenotype cells, and CXCR3^+^CCR4^–^ Th1 cells. These phenotypes were quite variable between individual subjects (Fig. 4C). In contrast, previously hospitalized subjects demonstrated lower percentages of all three cell fates at early convalescent time points, indicating suboptimal SARS-CoV-2-specific CD4^+^ T cell responses despite the higher peak antibody levels in these subjects (Fig. 1J). A CXCR3^–^CCR4^+^ population was also detectable in all subjects, though it was not significantly different between non-hospitalized and previously hospitalized subjects. We also assessed bulk CD4^+^ T cells from the tetramer^–^ fraction as a control and found no differences in percentages of cTfh, activated cTfh, or CXCR3^–^CCR4^+^ populations, however we did detect an increased proportion of CXCR3^+^CCR4^–^ Th1 cells in non-hospitalized subjects.

Across all longitudinal subjects, we also observed some differences in the decay kinetics between the frequencies of SARS-CoV-2-specific subsets per million CD4^+^ T cells. The PD-1^hi^CXCR5^+^ cells exhibited the greatest average decay rate (*t*_1/2_ of 85 days) (Fig. 4C) as these cells presumably lost PD-1 expression and transitioned to memory PD-1^lo^CXCR5^+^ cells over time (*26*). Total CXCR5^+^ memory cTfh cells persisted more stably (*t*_1/2_ of 139 days) (Fig. 4D), as did CXCR3^+^CCR4^–^ memory Th1 cells (*t*_1/2_ of 102 days) (Fig. 4E) and CXCR3^–^CCR4^+^ cells (*t*_1/2_ of 130 days) (Fig. 4F). While we observed phenotypic differences in SARS-CoV-2-specific CD4^+^ T cells between non-hospitalized and previously hospitalized subjects at early memory time points, we were underpowered to directly assess whether this is a long-lasting feature due to limited longitudinal samples from previously hospitalized subjects.

### Associations between SARS-CoV-2 epitope-specific CD4^+^ T cell and antibody responses

**Fig. 5.**
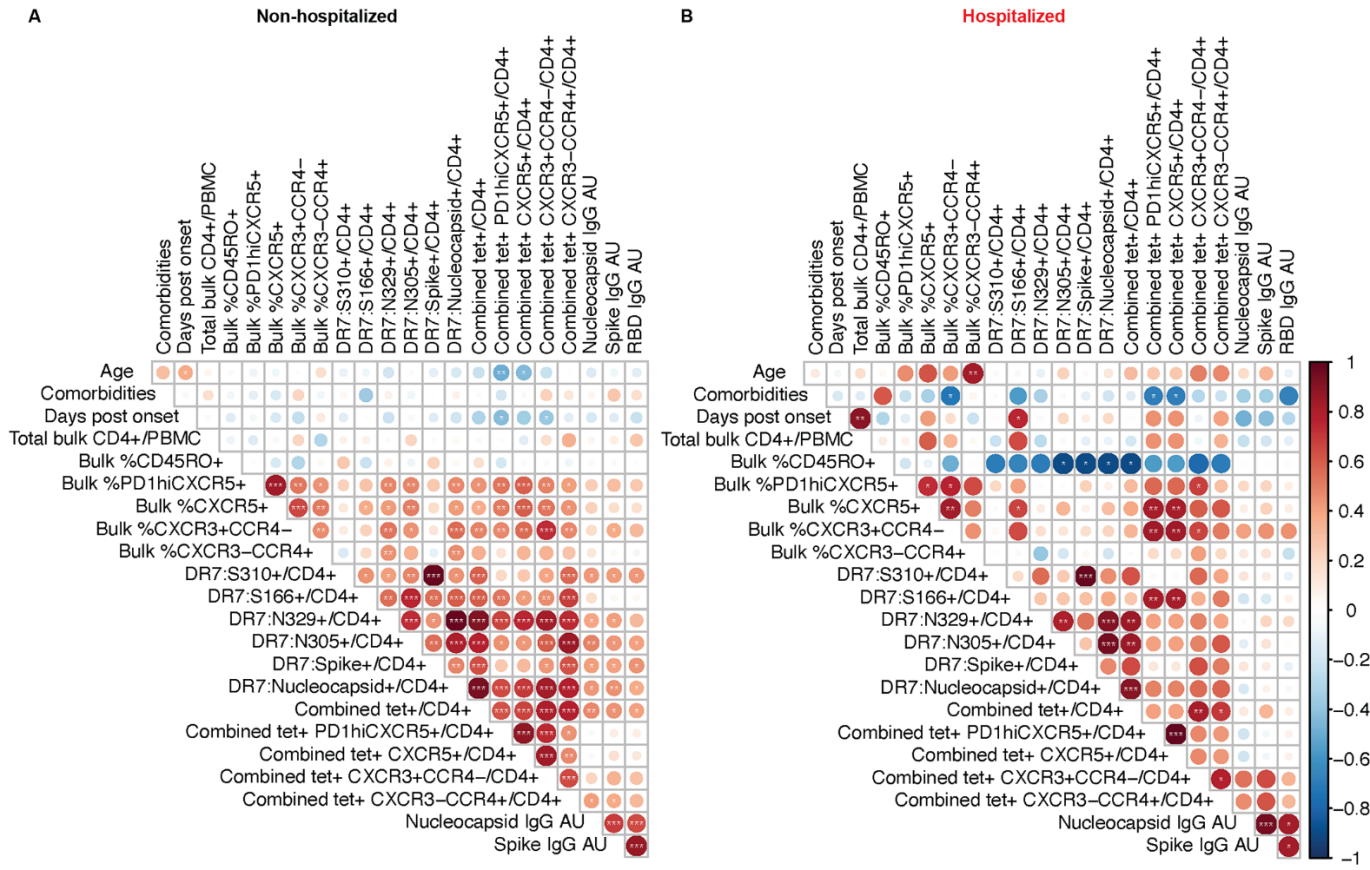
Associations between SARS-CoV-2 epitope-specific CD4^+^ T cell and antibody responses. (**A-B**) Correlation matrices from non-hospitalized (n = 31) (**A**) or previously hospitalized (n = 9) (**B**) subjects displaying Spearman rank order correlation values indicated by circle size and color from red (1) to blue (-1) and p values indicated by white asterisks. *p < 0.05, **p <0.01, ***p < 0.001. Data represents the first time point of collection for each subject (ranging from day 13 to day 181 post symptom onset). Most subjects had all parameters measured, but a few subjects had 1-2 data points that were not collected (as detailed in Table S4). Comorbidity indices were defined by the number of conditions considered high risk for severe disease as listed by the Centers for Disease Control and Prevention.

To broadly assess relationships between CD4^+^ T cell and antibody responses following COVID-19, we correlated individual parameters for all first time point blood draws by pairwise comparisons. For this analysis, we again segregated non-hospitalized (Fig. 5A) and previously hospitalized (Fig. 5B) subjects to partially control for differences in disease severity in our assessment.

There was an inverse relationship between the proportion of CD45RO^+^ antigen-experienced cells and the frequency of SARS-CoV-2-specific CD4^+^ T cell responses per million CD4^+^ T cells in previously hospitalized subjects but not in subjects with a history of mild COVID-19 (non-hospitalized). These results are consistent with the finding that low frequency of naïve T cells is a strong predictor of COVID-19 disease severity (*13*). This correlation is also of interest given that mouse studies have shown that absolute number of naïve CD4^+^ T cells positively correlates with the size of pMHCII epitope-specific CD4^+^ T cell responses (*22*), and epitope-specific CD4^+^ T cell responses are critical for vaccine-induced protection to SARS strains (*27*). A non-continuous comorbidities index applied to each study subject also demonstrated a negative correlation with SARS-CoV-2-specific PD-1^hi^CXCR5^+^ activated cTfh and CXCR5^+^ cTfh cell frequency per million CD4^+^ T cells, as well as the percentage of bulk CD4^+^ T cells expressing the CXCR3^+^CCR4^–^ Th1 phenotype in previously hospitalized subjects but not in non-hospitalized subjects. In non-hospitalized subjects, age was inversely correlated with SARS-CoV-2-specific PD-1^hi^CXCR5^+^ activated cTfh and CXCR5^+^ cTfh cells.

This analysis also revealed modest correlations between some SARS-CoV-2 epitope-specific CD4^+^ T cell populations and antibody titers. In particular, SARS-CoV-2-specific cTfh frequency per million CD4^+^ T cells did not clearly correlate with the magnitude of early antibody responses in either group. This was not unexpected, given the previous findings that the highest titer antibodies (Fig. 1J) were in hospitalized patients, who also demonstrated poor cTfh cell generation (Fig. 4C). Taken as a whole, milder cases of COVID-19 in the non-hospitalized group resulted in stronger correlations between SARS-CoV-2-specific CD4^+^ T cell responses and antibody responses, suggesting more coordinated cellular and humoral immunity.

While cTfh populations were not highly correlated with peak antibody responses, we hypothesized they could be more related to antibody stability through the generation of long-lived plasma cells. To determine whether linked recognition of T cell and B cell epitopes could account for differences in the stability of anti-S, anti-RBD, and anti-N antibody responses noted in Fig. 3, we also compared spike (DR7:S310 and DR7:S166)- and nucleocapsid (DR7:N329 and DR7:N305)-specific cell phenotypes within individual subjects (Fig. S3). However, we did not find significant differences between spike- and nucleocapsid-specific activated cTfh or cTfh percentages, despite an increased proportion of spike-specific CXCR3^–^CCR4^+^ cells as compared to nucleocapsid-specific CXCR3^–^CCR4^+^ cells.

### Increased cTfh responses associated with sustained antibody responses

As stated, a subset of individuals within our longitudinal MassCPR cohort had mild symptoms and sustained antibody responses at or above the initial level for the duration of our study (up to 333 days post symptom onset). This antibody ‘sustainer’ phenotype was previously described in an earlier study of convalescent subjects enrolled in the BWH cohort (*9*). Antibody sustainers in the BWH cohort also had mild symptoms as a whole and shorter duration of symptoms when compared to individuals with decaying antibodies over time. Another key finding from this previous work was antibody sustainers also had evidence of increased early somatic hypermutation, which suggested germinal center dependence, yet analysis of bulk CD4^+^ T cells in these individuals did not find increased cTfh percentages in peripheral blood. Human cTfh cells reflect GC-dependent processes in other viral infections, such as the association between cTfh frequency and broadly neutralizing antibodies in HIV-infected individuals (*28*).

**Fig. 6.**
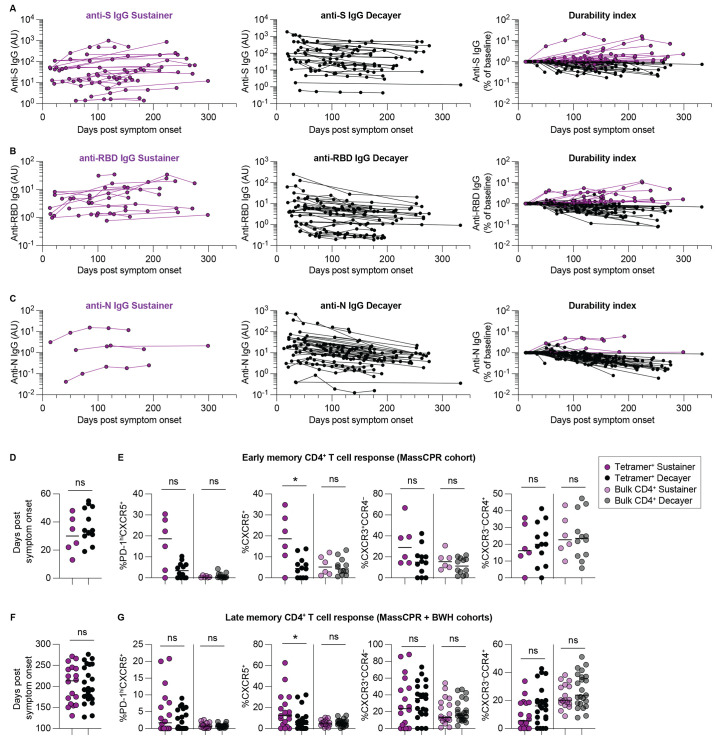
Increased cTfh responses associated with sustained antibody responses. (**A-C**) Longitudinal analysis illustrating changes in anti-S IgG (**A**), anti-RBD IgG (**B**) and anti-N IgG (**C**) antibodies over time from combined MassCPR and BWH cohorts (n = 53). Left column illustrates antibody sustainers (purple), middle column illustrates antibody decayers (black), and right column indicates antibody durability indices of both sustainers and decayers. (**D**) Comparison of timing of sample collection of PBMC from antibody sustainers (n = 6) and antibody decayers (n = 12) for CD4^+^ T cell phenotype comparisons at early convalescent time points. (**E**) Percentages of PD-1^hi^CXC5^+^, CXCR5^+^, CXCR3^+^CCR4^–^ and CXCR3^–^CCR4^+^ positive cells within combined tetramer^+^ and tetramer^–^ bulk CD4^+^ T cells from sustainers and decayers at early convalescent time points. (**F**) Comparison of timing of sample collection of PBMC from antibody sustainers (n = 18) and antibody decayers (n = 23) for phenotypic comparisons at late convalescent time points. (**G**) Percentages of PD-1^hi^CXC5^+^, CXCR5^+^, CXCR3^+^CCR4^–^ and CXCR3^–^CCR4^+^ positive cells within combined tetramer^+^ and tetramer^–^ bulk CD4^+^ T cells from sustainers and decayers at late convalescent time points. Horizontal lines indicate median values. Statistics by Mann-Whitney tests. *p < 0.05. ns = not statistically significant.

We identified an additional 21 HLA-DR7^+^ individuals in the BWH cohort described in Chen *et al*. (*9*). Figure 6A-C demonstrates the longitudinal antibody responses (all time points) among both MassCPR and BWH cohorts. Across the pooled cohorts, most sustained antibody responses targeted the S antigen. Individuals were grouped as antibody sustainers or decayers based upon association with an anti-S or anti-RBD antibody durability index >1 or <1, at the final time point of collection.

We next compared SARS-CoV-2-specific CD4^+^ T cell responses of sustainers versus decayers at ‘early’ convalescent time points where PBMC samples were available within the MassCPR cohort. Figure 6D illustrates the timing of these samples, within 60 days of symptom onset. Sustained antibody production was associated with increased CXCR5^+^ memory cTfh cells as a percentage of all SARS-CoV-2-specific cells detected with tetramers (Fig. 6E). To assess whether this persisted, we analyzed CD4^+^ T cell responses at the last time point of antibody collection within the combined MassCPR and BWH cohort subjects (ranging from 127 to 276 days after symptom onset). The timing of these ‘late’ convalescent samples from both cohorts are summarized in Fig. 6F. At these later time points, we found a similar result, suggesting long term persistence of SARS-CoV-2-specific memory cTfh cells in antibody sustainers (Fig. 6G).

## DISCUSSION

This study focuses on the relationship between SARS-CoV-2-specific CD4^+^ T cells and antibody responses that persist following COVID-19, which were assessed during the first wave of infections in Boston, Massachusetts, United States, in individuals prior to vaccination. We confirmed several previously described aspects of humoral immunity: peak antibody levels were associated with increased infection severity; the rate of decay was different for anti-S, anti-RBD, and anti-N IgG antibodies; and a subset of individuals with mild COVID-19 disease course had sustained antibody responses that remained stable or rising for the duration of the study.

We utilized a peptide:MHCII tetramer-based enrichment strategy to analyze CD4^+^ T cell responses to two DR7:S epitopes and two DR7:N epitopes. Our analysis found different CD4^+^ T cell epitope-specific responses had relatively stable half-lives between ~4-6 months. SARS-CoV-2-specific CD4^+^ T cell response frequencies were not significantly different between cases of mild (non-hospitalized) and moderate to severe (previously hospitalized) COVID-19 when normalized to total CD4^+^ T cells in circulation, though the trend appeared to be toward increased frequencies of SARS-CoV-2-specific cells in the more severe cases. Taken into the context of CD4^+^ T cell lymphopenia that occurs in severe COVID (*29*–*32*), it is unclear how the frequencies of circulating SARS-CoV-2-specific cells per million CD4^+^ T cells relate to absolute cell number. The number of SARS-CoV-2-specific cells per unit of blood may be a better proxy of total cell numbers, however this analysis was not possible in this study due to the differing sample acquisition methods used, either from peripheral blood draw or leukapheresis.

We did however detect significant phenotypic differences among SARS-CoV-2-specific cells across disease severities. The lack of cTfh responses from patients previously hospitalized with moderate to severe COVID-19 was consistent with the decreased germinal centers observed in the lymph nodes of patients with severe or fatal infection (*10*). Together these findings could suggest that severe infection with SARS-CoV-2 elicits primarily T-independent or extrafollicular B cell responses and perhaps lower affinity and shorter-lived B cell responses consequently. Higher peak antibody responses in individuals with a severe disease course could be related to other factors not assessed, such as viral load, than to CD4^+^ T cell “help.”

We also found a relationship between antibody durability and percent of SARS-CoV-2-specific cells with a cTfh phenotype that persisted into the late memory phase, which was not previously appreciated without methods for directly identifying SARS-CoV-2-specific responses (*9*). While not assessed here, additional metrics of high-quality B cell responses following COVID-19, such as somatic hypermutation and memory B cell formation may also be dependent upon coordinated CD4^+^ T cell responses. CD4^+^ T cells primed in the setting of severe COVID-19 also appeared to have impaired Th1 memory formation, based upon decreased percentages of circulating CXCR3^+^CCR4^–^ cells at early memory time points. This was surprising based upon earlier studies suggesting that Th1 cells are the cellular source for abundant TNF or other cytokines that could suppress GC formation during acute infection (*10*). We suspect CXCR3^–^CCR4^+^ cells are an indicator of lung homing following COVID-19 (*33*), however we did not assess Th2 cytokines due to the low frequency of tetramer^+^ cells.

Our pMHCII tetramer-based analysis did not find evidence for strong pre-existing immunity to the 2 spike and 2 nucleocapsid CD4^+^ T cell epitopes assessed. It should be noted, however, that these epitopes do not have significant sequence homology with common cold coronaviruses, which may account for the lack of significant pre-existing T cell immunity detected in unexposed subjects, as has been documented with larger-scale screens of SARS-CoV-2 CD4^+^ T cell epitopes using different methods (*17*, *21*, *34*–*36*). Tetramers may also be less likely to pick up lower affinity cells with a higher degree of cross-reactivity across coronavirus strains detected by AIM assays (*37*). This could also influence the phenotypic differences we observed between mild and severe disease, given previous findings suggesting lower affinity cells comprise a larger proportion of the SARS-CoV-2-specific CD4^+^ T cell response in severe disease (*38*). Further studies using a combination of CD4^+^ T cell detection methods in concert may help clarify the relative contributions of high and low affinity cells to infection- and vaccine-induced immunity.

Clustering patients based upon hospitalization status as an indicator of disease severity has potential caveats, including immune factors such as immunomodulatory therapies and non-immune factors such as comorbidities, that may contribute to illness without directly influencing immune cell function. The decreased number of previously hospitalized subjects compared to non-hospitalized subjects was also a limitation of this study. Notably, none of the study subjects had record of a defined immunodeficiency in their medical history. Immunomodulatory therapies received during acute infection could certainly affect long term T and B cell immunity, though we were underpowered to assess this as only 3 hospitalized subjects in the present study received such treatments.

In conclusion, our study demonstrates the usefulness of directly tracking SARS-CoV-2-specific responses with pMHCII tetramers, an approach that has been underutilized for characterizing correlates of SARS-CoV-2 immunity. Our results add to the growing body of evidence for immune dysfunction in severe COVID-19. Overall, these data suggest individuals with a less severe disease course generate stronger Tfh differentiation and more durable, GC-dependent high-affinity antibodies produced by long-lived plasma cells. Memory cTfh responses also persist several months after resolution of primary infection. This is potentially very advantageous to the host, who may be better equipped to re-engage germinal centers and generate new antibodies with higher affinity and breadth with subsequent exposures, including to variant virus strains.

## MATERIALS AND METHODS

### Study Design

This study aimed to better understand long-term immunity following COVID-19. We generated peptide:MHCII tetramers to analyze SARS-CoV-2-specific CD4^+^ T cell responses to spike and nucleocapsid epitopes, along with paired antibody responses, from peripheral blood samples in longitudinal cohorts of human subjects across a range of disease severities. All subjects were recruited with informed consent, and the study was approved by the Mass General Brigham Institutional Review Board. For each individual, basic demographic information including age and sex, as well as medical history and COVID-19 history were obtained, as summarized in Table S2.

### Human blood samples

Peripheral blood samples from convalescent subjects were drawn into EDTA (Ethylenediamine Tetraacetic Acid) tubes or collected through a leukapheresis procedure. PBMC were isolated by Ficoll-Hypaque density gradient centrifugation and cryopreserved in fetal bovine serum (FBS) containing 10% dimethyl sulfoxide (DMSO). Pre-pandemic controls were collected prior to December 2019 at MGH or purchased commercially (STEMCELL Technologies).

### Tetramer generation

The generation of peptide:MHCII tetramers has been described in detail (*39*, *40*). Soluble versions of HLA-DRB1 molecules (intracellular and transmembrane domains truncated) were covalently linked to SARS-CoV-2 peptide epitopes through a glycine-serine linker were co-expressed in *Drosophila* S2 cells with a soluble HLA-DRBA*01:01 construct containing a C-terminal BirA biotinylation site sequence, the BirA biotin ligase enzyme to mediate in vivo biotinylation of the recombinant protein, and a puromycin drug resistance gene. Efficient heterodimerization of the HLA-DR chains was stabilized by complementary Fos-Jun leucine zipper motifs engineered into the constructs. After puromycin selection and scale up, cell supernatants were harvested and biotinylated peptide:MHCII complexes were purified via immunoaffinity chromatography with an antibody to HLA-DR (clone L243). The extent of peptide:MHCII complex biotinylation was determined empirically by direct titration (visualized by Western blot) to PE, APC, or PE-Cy7 fluorochrome-conjugated streptavidin reagents (Prozyme or Invitrogen). Biotinylated peptide:MHC complexes were then mixed in excess of a 4:1 stoichiometric ratio to streptavidin conjugates, filtered to remove aggregates, and concentrations were calculated and adjusted by measuring optical density of the streptavidin fluorochromes.

### Tetramer enrichment and flow cytometry

Cryopreserved samples containing 1-10 × 10^7^ PBMC from DRB1*07:01 (DR7)^+^ convalescent subjects or pre-pandemic negative controls were thawed, washed twice with PBS containing 2% FBS, and resuspended in either complete EHAA or RPMI 1640 with 5% FBS. Staining with a pre-mixed cocktail of DR7:p-streptavidin-PE, DR7:p-streptavidin-PE-Cy7, and DR7:p-streptavidin-APC tetramers at a final concentration of 10 nM for each reagent was for 1 hour at room temperature. Anti-PE and anti-APC magnetic beads (Miltenyi Biotec) were then added, and bead-bound cells were enriched as previously described (*22*). Cells in the bound and unbound fractions were stained with Zombie Aqua viability dye (BioLegend) and a surface marker antibody panel consisting of anti-CD20^BV510^ (clone 2H7, BioLegend), anti-CD14^BV510^ (clone M5E2, BioLegend), anti-CD3^AF700^ (clone UCHT1, BioLegend), anti-CD4^BV605^ (clone OKT4, BioLegend), anti-CD8^BUV395^ (clone RPA-T8, BioLegend), anti-CD45RO^APC/Cy7^ (clone UCHL1, BioLegend), anti-CXCR3^PE/Dazzle594^ (clone G025H7, BioLegend), anti-CXCR5^BV421^ (clone J252D4, BioLegend), anti-PD-1^BV785^ (clone EH12.2H7, BioLegend), and anti-CCR4^PerCP/Cy5.5^ (clone L291H4, BioLegend) for 30 min at room temperature. Data were acquired on an LSR Fortessa X-20 (BD) flow cytometer and analyzed with FlowJo software (BD).

### Calculation of tetramer binding cells per million (10^6^) CD4^+^ T cells

The total number of tetramer specific cells per sample was obtained by multiplying the number of single, live, tetramer-bound CD4^+^ T cells eluted from the anti-PE and anti-APC enrichment columns (bound fraction) by the quotient of the total number of cell count beads added to the bound cell fraction divided by the number of cell count beads detected in the bound fraction. To calculate the frequency of tetramer specific cells per million CD4^+^ T cells, the total number of tetramer specific cells in the sample was divided by the total number of live, CD4^+^ cells in the entire sample (bound and flow through) determined by cell count beads in both fractions and multiplied by 1 million.

### ELISA

Quantitation of plasma IgG reactive to SARS-CoV-2 spike, RBD, and nucleocapsid antigens was performed on convalescent COVID-19 subjects and healthy pre-pandemic controls (Collected before October 2019), as previously described (*9*). Antibody durability index was calculated as the quotient of the final draw IgG level divided by the 1st draw IgG level for each antigen.

### Statistical analysis

Data and statistical analyses were performed using FlowJo 10 and GraphPad Prism 9, with the exception of the Fig. 5 correlation matrix, which was generated using the corrplot package (v0.84) (*41*) in RStudio (v1.4.1106) running R (v4.0.4). The lowest detected frequency of SARS-CoV-2 epitope-specific CD4^+^ T cells was 0.6 per million CD4^+^ T cells, so we imputed the undetectable values shown on the graphs at that value. We set the limit of detection for SARS-CoV-2 epitope-specific CD4^+^ T cells at 0.9, which is the mean value for CD8^+^ T cells detected with HLA-DR restricted tetramers (Fig. S2). For longitudinal analyses, half-life calculations excluded any subjects from which only one time point was obtained or where values were undetectable at two or more time points, though all data were included in the graphs. A simple linear regression of log(2)-transformed cell frequency was performed for each longitudinal subject and the slopes compared by one-way ANOVA. The average *t*_1/2_ values reported were calculated as a function of 1/[average slope] of the log(2)-transformed data. Only samples that contained at least 10 total tetramer-bound cells were used for phenotypic comparisons (Figs. 4, 6 and S3). Mann-Whitney or Kruskal-Wallis with Dunn’s multiple comparisons tests were applied for unpaired comparisons and Wilcoxon tests were applied for paired comparisons, as indicated in the respective figure legends. Correlations were performed using pairwise Spearman rank order correlation.

## MGH COVID-19 Collection & Processing Team

Betelihem A. Abayneh, Patrick Allen, Galit Alter, Diane Antille, Katrina Armstrong, Alejandro Balazs, Julia Bals, Max Barbash, Yannic Bartsch, Julie Boucau, Siobhan Boyce, Joan Braley, Karen Branch, Katherine Broderick, Julia Carney, Andrew Chan, Josh Chevalier, Fatema Chowdhury, George Daley, Susan Davidson, Michael Dougan, David Drew, Kevin Einkauf, Ashley Elliman, Jon Fallon, Liz Fedirko, Kelsey Finn, Keith Flaherty, Jeanne Flannery, Pamela Forde, Pilar Garcia-Broncano, Elise Gettings, David Golan, Amanda Griffin, Sheila Grimmel, Kathleen Grinke, Kathryn Hall, Ciputra Hartana, Meg Healy, Howard Heller, Deborah Henault, Grace Holland, Chenyang Jiang, Nikolaus Jilg, Paulina Kaplonek, Marshall Karpell, Chantal Kayitesi, Evan C. Lam, Vlasta LaValle, Kristina Lefteri, Xiaodong Lian, Mathias Lichterfeld, Daniel Lingwood, Hang Liu, Jinqing Liu, Yuting Lu, Sarah Luthern, Natasha Ly, Jordan Marchewka (Schneider), Brittani Martino, Roseann McNamara, Ashlin Michell, Ilan Millstrom, Noah Miranda, Christian Nambu, Susan Nelson, Marjorie Noone, Claire O'Callaghan, Christine Ommerborn, Matthew Osborn, Lois Chris Pacheco, Nicole Phan, Shiv Pillai, Falisha A Porto, Yelizaveta Rassadkina, Alexandra Reissis, Alex Rosenthal, Francis Ruzicka, Edward Ryan, Kyra Seiger, Kathleen Selleck, Libera Sessa, Arlene Sharpe, Christianne Sharr, Sally Shin, Nishant Singh, Sue Slaughenhaupt, Kimberly Smith Sheppard, Weiwei Sun, Xiaoming Sun, Elizabeth Suschana, Hannah Ticheli, Alicja Trocha-Piechocka, Vivine Wilson, Colline Wong, Daniel Worrall, Alex Zhu, Zachary Manickas-Hill, Edward DeMers, Kelly Judge, Musie S. Ghebremichael, Peggy Lai, Jonathan Li, Xu G. Yu, Bruce Walker, Mary Carrington, Maureen Martin, Yuko Yuki
