# Supplementary material for: SARS-CoV-2 epitope-specific CD4+ memory T cell responses across COVID-19 disease severity and antibody durability
Source: Sci Immunol. 2022 Apr 21:eabl9464. doi: 10.1126/sciimmunol.abl9464 (PMC9097883; doi:10.1126/sciimmunol.abl9464)
Supplement: Supplementary file 1 — Figs. S1 to S3 Tables S1 and S2 [file sciimmunol.abl9464_sm.pdf]

Science Immunology
AAAS

Supplementary Materials for

SARS-CoV-2 epitope-specific CD4$^+$ memory T cell responses across COVID-19 disease severity and antibody durability

Ryan W. Nelson *et al.*

Corresponding author: Andrew D. Luster, aluster@mgh.harvard.edu

DOI: 10.1126/sciimmunol.abl9464

The PDF file includes:

Figs. S1 to S3
Tables S1 and S2

Other Supplementary Material for this manuscript includes the following:

Table S3

SARS-CoV-2 epitope-specific CD4$^+$ memory T cell responses across COVID-19 disease severity and antibody durability

Authors: Ryan W. Nelson,[1,2] Yuezhou Chen,[3] Olivia L. Venezia,[2,7] Richard M. Majerus,[4] Daniel S. Shin,[1,2] MGH COVID-19 Collection & Processing Team, Mary N. Carrington,[5,6] Xu G. Yu,[5,7] Duane R. Wesemann,[3,5] James J. Moon,[2,8] Andrew D. Luster[2*]

Affiliations:

[1]Division of Immunology, Boston Children's Hospital, Harvard Medical School; Boston, MA, USA.

[2]Center for Immunology and Inflammatory Diseases, Division of Rheumatology, Allergy and Immunology, Massachusetts General Hospital, Harvard Medical School; Boston, MA, USA.

[3]Department of Medicine, Division of Allergy and Clinical Immunology, Division of Genetics, Brigham and Women's Hospital, Harvard Medical School; Boston, MA, USA.

[4]Queens University of Charlotte, Charlotte, NC, USA.

[5]Ragon Institute of MGH, MIT and Harvard; Cambridge, MA, USA.

[6]Basic Science Program, Frederick National Laboratory for Cancer Research, National Cancer Institute, Frederick, MD and Laboratory of Integrative Cancer Immunology, Center for Cancer Research, National Cancer Institute; Bethesda, MD, USA.

[7]Infectious Disease Division, Brigham and Women's Hospital, Harvard Medical School; Boston, MA, USA.

[8]Division of Pulmonary and Critical Care Medicine, Massachusetts General Hospital, Harvard Medical School; Boston, MA, USA.

[*]Corresponding author. Email: aluster@mgh.harvard.edu

Supplementary Materials

Fig. S1. Gating strategy for tetramer enriched $CD4^+$ T cells and screening putative $CD4^+$ T cell epitopes in convalescent COVID-19 subjects.

Fig. S2. Determination of limit of detection based upon background $CD8^+$ T cell staining.

Fig. S3. Comparison of DR7:S- and DR7:N-$tetramer^+$ $CD4^+$ T cell phenotypes.

Table S1. Peptide sequences of tetramers tested in convalescent subjects.

Table S2. Subject characteristics.

Table S3. Raw data.

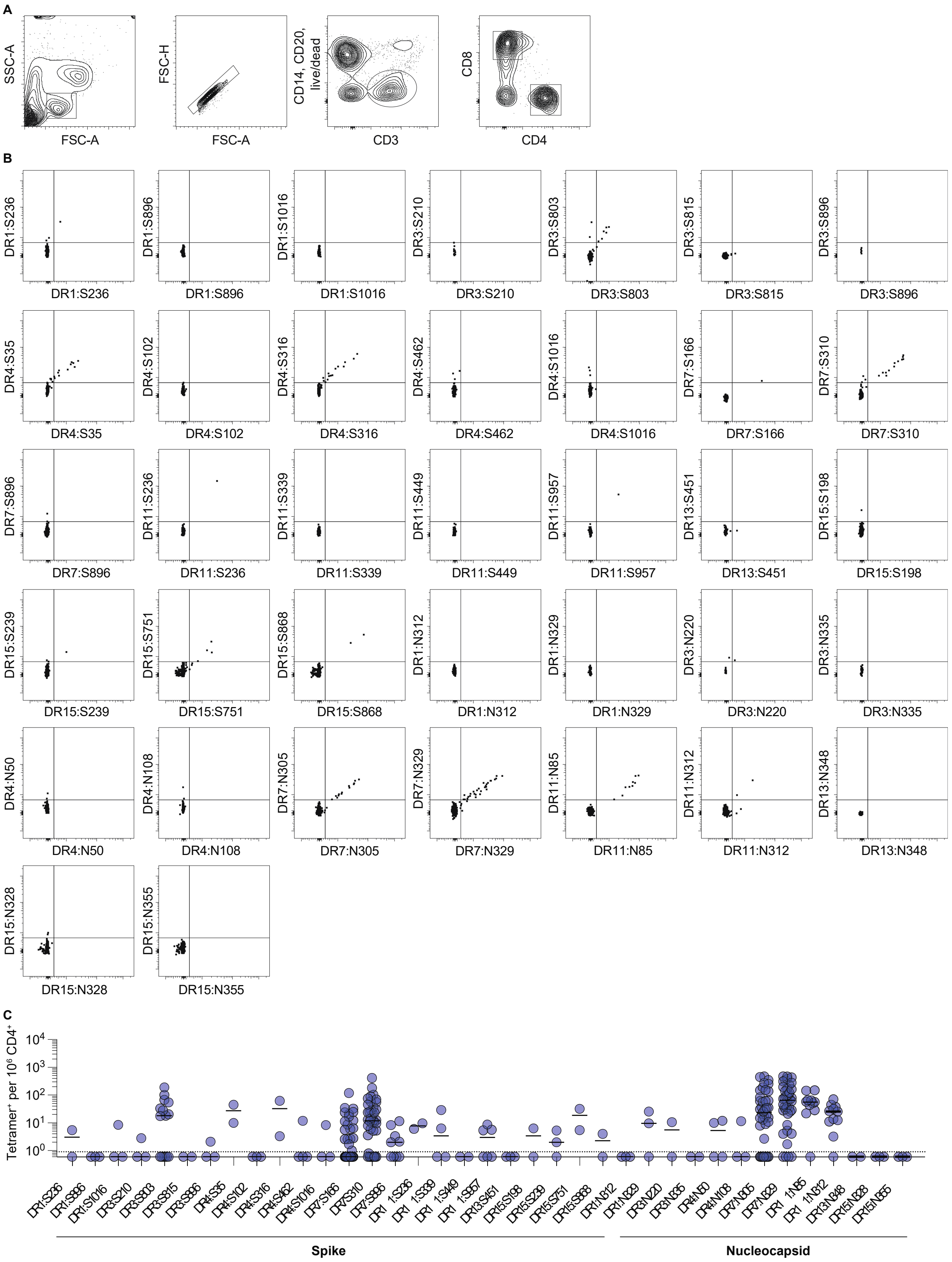

A
SSC-A
FSC-A
FSC-H
FSC-A
CD14, CD20, live/dead
CD3
CD8
CD4
B
C
Tetramer⁺ per 10⁶ CD4⁺
Spike
Nucleocapsid

Fig. S1. Gating strategy for tetramer enriched $CD4^+$ T cells and screening putative $CD4^+$ T cell epitopes in convalescent COVID-19 subjects. (**A**) Representative flow cytometry plots demonstrating gating strategy on live $CD14^-CD20^-CD3^+CD8^-CD4^+$ T cells following magnetic bead enrichment of tetramer bound cells. (**B**) Representative flow cytometry plots of dual PE and APC tetramer-stained cells from HLA-matched samples from convalescent COVID-19 subjects. (**C**) Summary of individual epitope-specific $CD4^+$ T cell frequencies per million $CD4^+$ T cells.

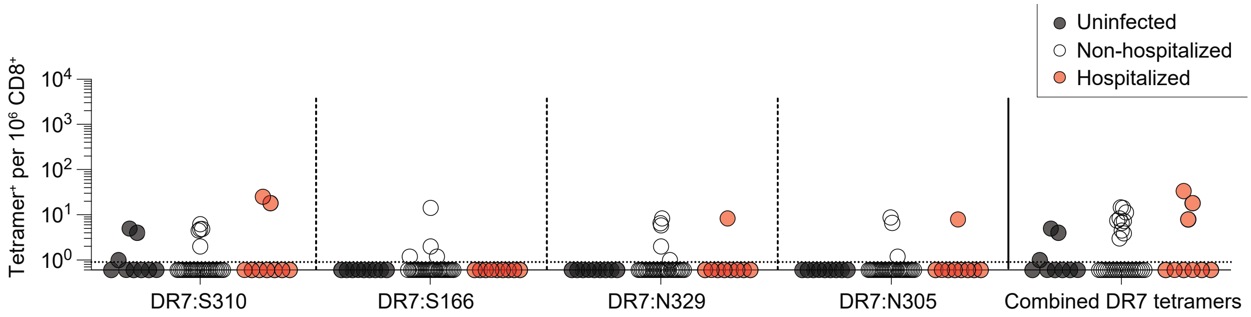

Fig. S2. Determination of limit of detection based upon background $CD8^+$ T cell staining.

Summary of frequencies of live $CD14^-CD20^-CD3^+CD8^+CD4^-$ T cells detected with HLA-DR7-restricted tetramers per million $CD8^+$ T cells.

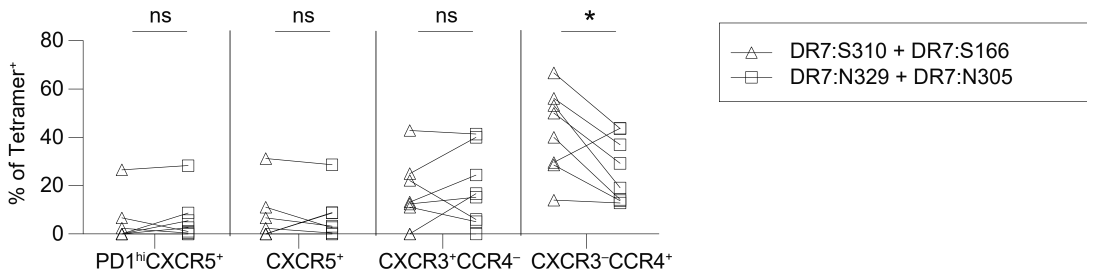

Fig. S3. Comparison of DR7:S- and DR7:N-tetramer^{+} CD4^{+} T cell phenotypes. Percentages of PD-1^{hi}CXC5^{+}, CXCR5^{+}, CXCR3^{+}CCR4^{-} and CXCR3^{-}CCR4^{+} positive cells within DR7:S- and DR7:N-tetramer^{+} CD4^{+} T cell populations. Lines indicate detection within the same sample. Statistics by Wilcoxon tests. *$p < 0.05$. ns = not statistically significant.

**HLA Allele**	**Peptide**	**Sequence**
Spike protein (S)		
DRB1*01:01	236-247	TRFQTLLALHRS
	896-906	IPFAMQMAYRF
	1016-1026	AEIRASANLAA
DRB1*03:01	210-221	INLVRDLPQGFS
	803-904	SQILPDPSKPSK
	815-826	RSFIEDLLFNKV
	896-908	IPFAMQMAYRFNG
DRB1*04:01	35-46	GVYYPDKVFRSS
	102-114	RGWIFGTTLDSKT
	316-327	SNFRVQPTESIV
	462-472	KPFERDISTEI
	1016-1026	AEIRASANLAA
DRB1*07:01	166-177	CTFEYVSQPFLM
	310-320	KGIYQTSNFRV
	896-906	IPFAMQMAYRF
DRB1*11:01	236-247	TRFQTLLALHRS
	339-350	GEVFNATRFASV
	449-460	YNYLYRLFRKSN
	957-967	QALNTLVKQLS
DRB1*13:01	451-461	YLYRLFRKSNL
DRB1*15:01	198-208	DGYFKIYSKHT
	239-249	QTLLALHRSYL

	751-762	NLLLQYGSFCTQ
	868-879	EMIAQYTSALLA
Nucleocapsid protein (N)		
DRB1*01:01	312-323	SAFFGMSRIGME
	329-340	TWLTYTGAIKLD
DRB1*03:01	220-231	ALLLLDRLNQLE
	335-346	GAIKLDDKDPNF
DRB1*04:01	50-61	ASWFTALTQHGK
	108-119	WYFYYLGTGPEA
DRB1*07:01	305-316	AQFAPSASAFFG
	329-340	TWLTYTGAIKLD
DRB1*11:01	85-96	GYYRRATRRIRG
	312-323	SAFFGMSRIGME
DRB1*13:01	348-359	DQVILLNKHIDA
DRB1*15:01	328-339	GTWLTYTGAIKL
	355-366	KHIDAYKTFPPT

Table S1. Peptide sequences of HLA-DR tetramers tested in convalescent subjects. HLA-DRB1*07:01 (DR7)$^{+}$ tetramers utilized in Figures 1-6 highlighted in blue.

**<u>MassCPR cohort</u> (n = 40 HLA-DR7^{+} subjects)**	
**Age (years)**	24 to 81 (median = 48.5, IQR* = 26.75)
**Sex**	
Male (%)	22.5% (9/40)
Female (%)	77.5% (31/40)
**Race**	
African American or Black (%)	5% (2/40)
American Indian or Alaska Native (%)	0% (0/40)
Asian (%)	5% (2/40)
Multiracial (%)	2.5% (1/40)
Native Hawaiian or Other Pacific Islander	0% (0/40)
Unknown (%)	5% (2/40)
White (%)	82.5% (33/40)
**Ethnicity**	
Hispanic or Latino (%)	7.5% (3/40)
Non-Hispanic (%)	92.5% (37/40)
**Hospitalization status**	
Never hospitalized (%)	77.5% (31/40)
Total hospitalized (%)	22.5% (9/40)
ICU admission (% of hospitalized)	67% (6/9)
**Sample collection dates**	April 2020-January 2021
**Days post-symptom onset at collection; n = 86**	13 to 299 (median = 119, IQR* = 141.25)
**Blood collection frequency**	
Multiple time point donors (2 to 3 times)	75% (30/40)
Single time point donors	25% (10/40)
**Treatment**	

Tocilizumab	2.5% (1/40)
Methylprednisolone	5% (2/40)
<u>**BWH cohort**</u> **(n = 21 HLA-DR7^{+} subjects)**	
**Age (years)**	23 to 71 (median = 40, IQR* = 24)
**Sex**	
Male (%)	29% (6/21)
Female (%)	62% (13/21)
Unknown (%)	10% (2/21)
**Race**	
African American or Black (%)	0% (0/21)
American Indian or Alaska Native (%)	0% (0/21)
Asian (%)	5% (1/21)
Multiracial (%)	0% (0/21)
Native Hawaiian or Other Pacific Islander	0% (0/21)
White (%)	95% (20/21)
**Ethnicity**	
Hispanic or Latino (%)	0% (0/21)
Non-Hispanic (%)	100% (21/21)
**Hospitalization status**	
Never hospitalized (%)	100% (21/21)
**New sample collection dates**	August-November 2020
**Days post-symptom onset at collection; n = 21**	150 to 242 (median = 177, IQR* = 42)

Table S2. Subject characteristics. *IQR, interquartile range.
